# Evidence for the Role of Epstein Barr Virus Infections in the Pathogenesis of Acute Coronary Events

**DOI:** 10.1371/journal.pone.0054008

**Published:** 2013-01-17

**Authors:** Philip F. Binkley, Glen E. Cooke, Amanda Lesinski, Mackenzie Taylor, Min Chen, Bryon Laskowski, W. James Waldman, Maria E. Ariza, Marshall V. Williams, Deborah A. Knight, Ronald Glaser

**Affiliations:** 1 Division of Cardiovascular Medicine, The Ohio State University College of Medicine, Columbus, Ohio, United States of America; 2 Department of Internal Medicine, Dorothy M. Davis Heart and Lung Research Institute, The Ohio State University College of Medicine, Columbus, Ohio, United States of America; 3 Department of Molecular Virology, Immunology and Medical Genetics, The Ohio State University College of Medicine, Columbus, Ohio, United States of America; 4 Institute for Behavioral Medicine Research, The Ohio State University College of Medicine, Columbus, Ohio, United States of America; 5 Department of Pathology, The Ohio State University College of Medicine, Columbus, Ohio, United States of America; Charite Universitätsmedizin, Germany

## Abstract

**Background:**

The role of viral infections in the pathogenesis of atherosclerosis remains controversial largely due to inconsistent detection of the virus in atherosclerotic lesions. However, viral infections elicit a pro-inflammatory cascade known to be atherogenic and to precipitate acute ischemic events. We have published in vitro data that provide the foundation for a mechanism that reconciles these conflicting observations. To determine the relation between an early viral protein, deoxyuridine triphosphate nucleotidohydrolase (dUTPase), produced following reactivation of Epstein Barr Virus (EBV) to circulating pro-inflammatory cytokines, intercellular adhesion molecule-1 (ICAM-1) and acute coronary events.

**Methodology/Principal Findings:**

Blood samples were obtained from 299 patients undergoing percutaneous coronary intervention for stable angina (SA), unstable angina (UA), or acute myocardial infarction (AMI). Plasma concentrations of pro-inflammatory cytokines and neutralizing antibody against EBV-encoded dUTPase were compared in the three patient groups. AMI was associated with the highest measures of interleukin-6 (ANOVA p<0.05; 4.6±2.6 pg/mL in patients with AMI vs. 3.2±2.3 pg/mL in SA). ICAM-1 was significantly higher in patients with AMI (ANOVA p<0.05; 304±116 pg/mL in AMI vs. 265±86 pg/mL SA). The highest values of ICAM-1 were found in patients having an AMI and who were antibody positive for dUTPase (ANOVA p = 0.008; 369±183 pg/mL in AMI and positive for dUTPase vs. 249±70 pg/mL in SA negative for dUTPase antibody).

**Conclusions/Significance:**

These clinical data support a model, based on in vitro studies, by which EBV may precipitate AMI even under conditions of low viral load through the pro-inflammatory action of the early protein dUTPase that is produced even during incomplete viral replication. They further support the putative role of viral infections in the pathogenesis of atherosclerosis and coronary artery events.

## Introduction

The role of viral infections in the pathogenesis of atherosclerosis has remained controversial. Although evidence from animal models and clinical studies has supported the atherogenic role of viral infections, other evidence has challenged this role in part due to the frequent inability to detect the culprit virus in atherosclerotic lesions [1]–[11]. However, evidence continues to accumulate in favor of the role of viral mediated atherosclerosis such as studies demonstrating that influenza vaccination can serve as secondary prevention for coronary events [11]–[15]. Recent evidence has shown a strong association between chronic infection with human papilloma virus and the incidence of coronary artery disease [16]. These associative studies prompt investigations that seek to provide insight into the mechanisms by which viral infection can exert a pro-atherogenic effect. We have published in vitro data that provide the foundation for a mechanism that reconciles the association between viral infections and the inability to detect significant viral loads in patients with atherosclerosis [17]. These data further provide a mechanistic framework by which at least a subset of viruses promotes the evolution of coronary artery disease.

The majority of evidence indicates that viral infections can promote both the evolution of atherosclerosis and the occurrence of acute coronary events through stimulation of the production and release of pro-inflammatory cytokines [3], [4], [6]–[9]. These cytokines as well as a variety of adhesion molecules are known to play a critical role in multiple phases of the evolution of atherosclerosis 18–30. We have shown that Epstein Barr Virus (EBV) encodes an enzyme, deoxyuridine triphosphate nucleotidohydrolase (dUTPase), as part of the synthesis of early proteins following reactivation of latent virus. dUTPase has been shown by our laboratory to induce peripheral blood monocytes to produce pro-inflammatory cytokines such as interleukin-6 (IL-6 ) and endothelial cell expression of intercellular adhesion molecule-1 (ICAM-1) [17], [31]–[34]. It is possible that EBV-encoded dUTPase induces a pro-inflammatory cascade even during incomplete viral replication when there is a low viral load that is difficult to detect using standard analytic methods. This would account for the inability of some reports to confirm the presence of a virus such as EBV despite its proposed role in atherogenesis.

In this investigation, we provide support for this proposed mechanism connecting EBV-encoded dUTPase, pro-inflammatory cytokines, intercellular adhesion molecules, and acute coronary events in the clinical setting.

## Methods

### Study Subjects

To determine whether the EBV-encoded dUTPase may play a role in coronary atherosclerosis and its major consequences, we enrolled 299 consecutive patients who were undergoing percutaneous coronary intervention (PCI) for symptomatic CAD, including stable angina (SA), unstable angina (UA), or acute myocardial infarction (AMI). All study subjects provided written informed consent and the protocol was approved by the Institutional Review Board for Human Subjects of The Ohio State University.

Patients were classified by CAD presentation (SA/elective, UA, or AMI) based on established clinical criteria and adjudicated by three cardiologists blinded to cytokine levels or anti-EBV-encoded dUTPase antibody status. MI was defined as evidence of myocardial necrosis with evidence of rise and/or fall of cardiac biomarkers with at least one value 3× the upper limit of normal (ULN) in addition to evidence of myocardial ischemia including symptoms of ischemia, ECG changes indicative of new ischemia (new ST-T changes or new left bundle branch block), or development of pathological Q waves on the ECG. ST segment elevation MI was differentiated from NSTEMI by the presence of greater than 1 mm ST segment elevation (injury current) on ECG in at least two contiguous leads. Unstable angina was defined as new onset angina occurring at rest or episodes similar to previous anginal episodes which are now prolonged or accelerated as reflected by an increase in severity, duration, frequency or requiring increased use of anti-angina therapy. ST-segment changes on the ECG were not mandatory and cardiac enzymes, by definition, could not be elevated.

All patients demonstrated a primary indication for PCI, including symptomatic disease with at least 75% angiographic luminal stenosis. SA/elective PCI patients served as controls.

### Serum Acquisition

Blood was obtained from enrolled patients at the time of the PCI procedure from the side-port of the femoral arterial sheath. One 8.5 ml serum separator tube (BD Vacutainer) was allowed to clot for 30 minutes post draw and then centrifuged for 15 minutes. Serum was then collected and stored at −80° F until time of use.

### Antibody titers to EBV-encoded dUTPase

To determine whether the EBV-encoded dUTPase and thus EBV may play a role in coronary atherosclerosis, we measured antibody levels to the EBV-encoded dUTPase using a modification of a previously described protocol [35]. Briefly, 5 µl of human serum was mixed with 5 µl of purified EBV-encoded dUTPase (3–5 units of enzyme) for 30 min at room temperature prior to assaying for dUTPase activity. dUTPase activity was determined using the standard disc assay [33]. The reaction mixture contained in a total volume of 0.1 ml: (50 mM Tris-HCl, pH 7.5, 0.1% bovine serum albumin, 1 mM MgCl_2_, 2 mM β-mercaptoethanol, 2 mM p-nitrophenyl phosphate, 5 mM ATP, 0.1 mM dUTP and 2 µCi [^3^H] dUTP) and incubated at 37°C for 1 hour. The reaction was stopped by spotting 50 µl of the reaction mixture onto a DE 81 filter disc, which was immediately placed into 18.4% formic acid solution. The discs were washed twice for 5 minutes each in the 18.4% formic acid solution followed by a five-minute wash in 95% ethanol. Next, the discs were dried and the amount of radioactivity, which represents non-hydrolyzed dUTP was determined by counting on a Beckman LS6000IC liquid scintillation counter. A unit was defined as the amount of enzyme that converts dUTP into 1 nmol dUMP per minute per ml under the assay conditions. For positive controls, assays were performed in the absence of serum and negative controls were performed in the absence of EBV-encoded dUTPase. Units neutralized were obtained as follows: (U_control_−U_serum_). Serum with neutralizing units greater than zero are considered “positive” for dUTPase neutralizing antibodies. Performance of the assays was done in a blinded manner, without knowledge of the patient's clinical presentation. For ease of description, patients who were found to have antibody to EBV encoded dUTPase will hereafter be referred to as “dUTPase positive.”

### Cytokine Measurement

ICAM-1, IL-6, IL-10, MCP-1/CCL2, CD40L, and TNFα concentrations were determined for each study subject using immunoassay (R&D Systems, Minneapolis, MN). Reagents and standards were prepared and assays performed per manufacture's instructions. Patient serum samples were utilized for each assay and were performed in duplicate for each cytokine. Performance of the assays was done in a blinded manner, without knowledge of the patient's clinical presentation.

### Statistical Methods

All continuous data are presented as the mean ±1 standard deviation. Baseline clinical characteristics were analyzed by factorial ANOVA for continuous variables and the Chi square test was used to compare nominal variables in the three clinical CAD presentation groups. Factorial ANOVA was also utilized to compare cytokine levels as the outcome measure in the three clinical CAD presentation groups. Classification of the clinical CAD presentation and presence or absence of antibody to dUTPase were independent factors. A p-value≤0.05 was considered to be statistically significant.

## Results

The baseline characteristics of study subjects are listed in Table 1. A total of 299 subjects who presented consecutively for PCI treatment for symptomatic CAD were enrolled. There were 145 subjects with SA (elective PCI), 92 subjects with UA, and 62 AMI subjects. Subjects were predominately male sex and Caucasian race, but were not significantly different between clinical presentation groups. A statistically significant difference in past history of CAD (74% in SA, 64% in UA, 45% in AMI; p<0.001) and hypertension ( 69% in SA, 75% in UA, 42% in AMI; p<0.001) was noted among groups. No other clinical differences between study groups were identified. In the AMI group, 63% were identified as having non ST-segment elevation MI and 37% as ST-segment elevation MI.

**Table 1:** Patient Characteristics.

	Stable Angina/Elective	Unstable Angina	Acute MI	p Value
	(N = 145)	(N = 92)	(N = 62)	
Age (yrs)	63.5±10.6	64.6±12.3	58.2±13.2	NS
(mean ± 1 SD)				
Male Sex (%)	96 (66%)	56 (61%)	41 (66%)	NS
Caucasian Race (%)	129 (89%)	84 (91%)	55 (89%)	NS
PMH				
CAD	108 (74%)	59 (64%)	28 (45%)	<0.001
MI	46 (32%)	28 (30%)	17 (27%)	NS
Hypertension	100 (69%)	69 (75%)	26 (42%)	<0.001
Hyperlipidemia	102 (70%)	66 (72%)	36 (58%)	NS
Diabetes Mellitus	54 (37%)	36 (39%)	22 (35%)	NS
FMH	66 (46%)	38 (41%)	35 (56%)	NS
Tobacco Use	81 (56%)	54 (59%)	29 (47%)	NS
MI at Presentation			62 (100%)	
NSTEMI			39 (63%)	
STEMI			23 (37%)	

Abbreviations: CAD = Coronary Artery Disease, FMH = Family Medical History for CAD, MI = Myocardial Infarction, NS = Non-significant, NSTEMI = Non-ST Segment Elevation Myocardial Infarction, PMH = Past Medical History, STEMI = ST Segment Elevation Myocardial Infarction.

**Figure 1:**
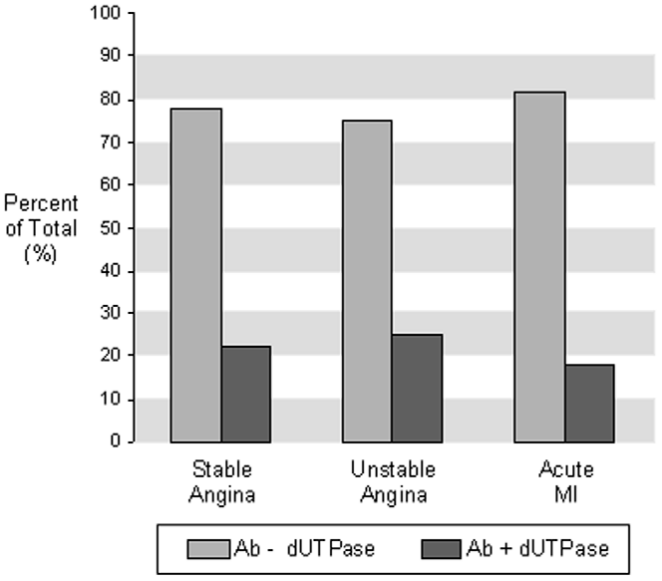
Frequency of the presence of the anti-EBV-encoded dUTPase antibody in the patient groups.

**Figure 2:**
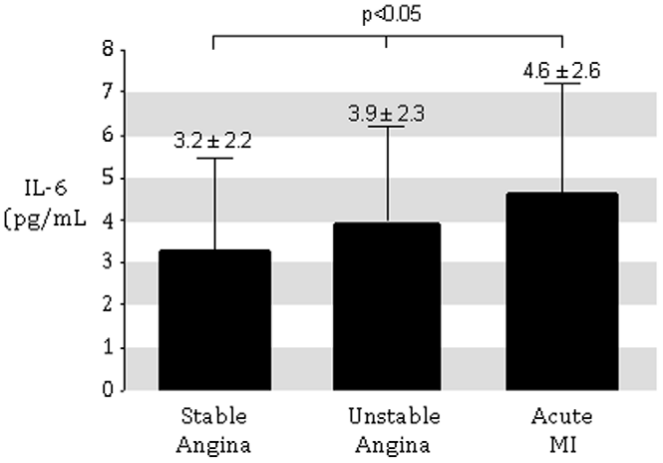
IL-6 concentrations in patients presenting with either SA, UA, or acute MI. SA/elective patients served as controls and demonstrated the lowest levels and acute MI patients had the highest levels. UA patients had intermediate levels.

**Figure 3:**
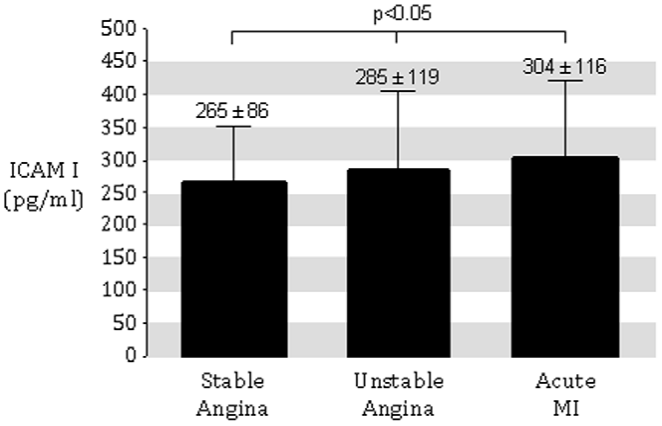
ICAM-1 concentrations in patients presenting with either SA, UA, or acute MI. SA/elective patients served as controls and again demonstrated the lowest levels while acute MI patients had the highest levels. UA patients had intermediate levels.

All 299 subjects were examined for the anti-EBV-encoded dUTPase antibody. There was no statistical difference in the frequency of dUTPase positive patients in the different groups (SA: n = 32 = 22%, UA: n = 23 = 25%, AMI : n = 11 = 18%; Figure 1). Acute myocardial infarction was associated with the highest measures of IL-6 compared to UA patients or SA/elective patients (4.6±2.6 pg/mL vs. 3.9±2.3 pg/mL vs. 3.2±2.3 pg/mL, respectively; p<0.05; Figure 2). Similarly, ICAM-1 was significantly higher in patients with AMI compared to UA patients or SA/elective patients (304±116 pg/mL vs. 285±119 pg/mL vs. 265±86 pg/mL, respectively; p<0.05; Figure 3).

**Figure 4:**
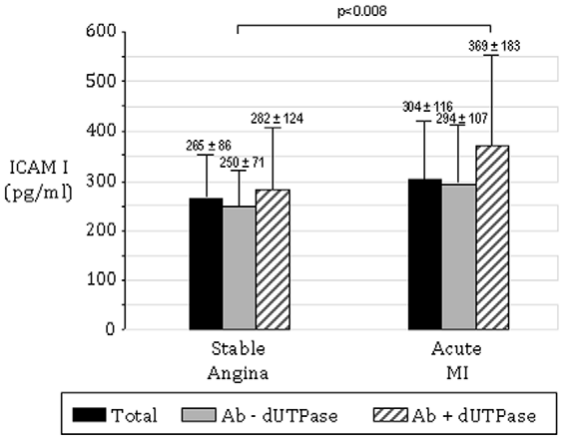
Means and standard deviations of ICAM-1 concentrations in patients presenting with either SA, or acute MI as a function of the presence of anti-EBV-encoded dUTPase antibody. The mean ICAM-1 concentrations for all patients in the SA and acute MI groups are shown in the far left bars. Patients with acute MI and positive for anti-EBV-encoded dUTPase antibody demonstrated the highest concentration of ICAM-1 while the lowest level was demonstrated in SA patients who were negative for anti-EBV-encoded dUTPase antibody (369±183 pg/mL vs. 249±70 pg/mL, respectively; p<0.008).

A significant relation was found between circulating concentrations of ICAM-1 and detectable antibody to EBV encoded dUTPase. The highest values of ICAM-1 were found in patients having an AMI and who were dUTPase positive (369±183 pg/mL in dUTPase positive patients with AMI and 249±70 pg/mL in dUTPase negative SA patients; p<0.008; Figure 4). The significant impact of dUTPase positive status is further shown through examination of hierarchical ANOVA models. Specifically, the overall significance of the ANOVA model increased from a p value of 0.01 for the univariable model with coronary artery disease status alone to 0.008 with the addition of dUTPase status to the model. Therefore, dUTPase status plays a significant role in explaining elevations in circulating ICAM-1.

**Figure 5:**
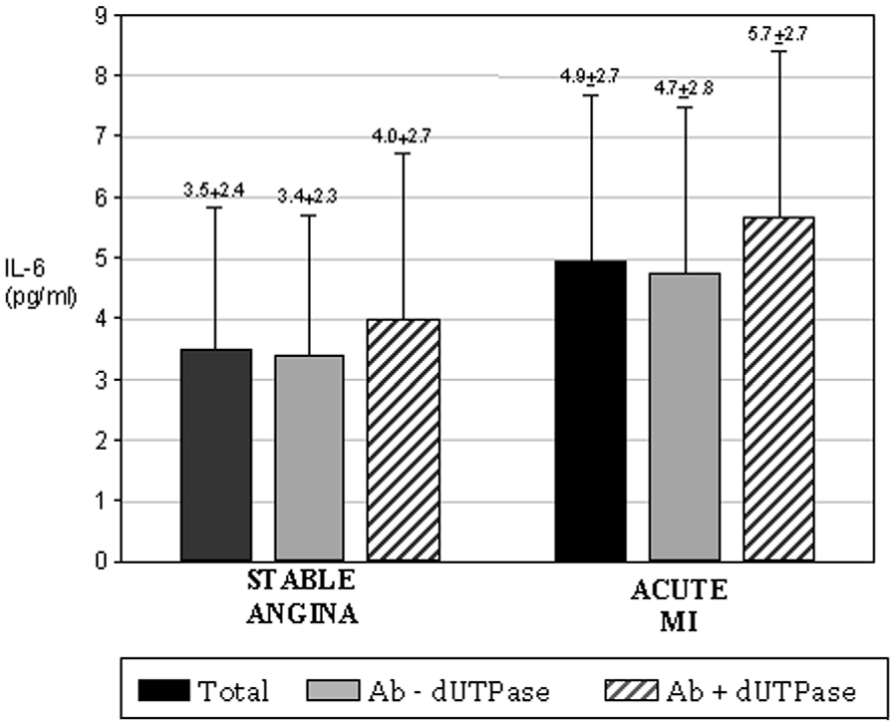
Means and standard deviations of IL-6 concentrations in patients presenting with either SA or acute MI as a function of the presence of anti-EBV-encoded dUTPase antibody. The mean IL-6 concentrations for all patients in the SA and acute MI groups are shown in the far left bars. Patients with acute MI and positive for anti-EBV-encoded dUTPase antibody demonstrated the highest concentration of IL-6 while the lowest level was demonstrated in SA patients who were negative for anti-EBV-encoded dUTPase antibody.

Although not achieving statistical significance, the relation between IL-6 and dUTPase showed a similar trend. The highest values for IL-6 were obtained in patients who had a myocardial infarction and were dUTPase positive (Figure 5).

No significant associations were found between IL-10, MCP-1/CCL2, CD40L, or TNFα concentrations and clinical presentation (data not shown).

## Discussion

This investigation provides essential clinical corroboration for a mechanism that accounts for the long-reported association between viral infections and coronary artery disease. Although a variety of studies have clearly demonstrated an association between atherosclerosis and viruses including cytomegalovirus, influenza and most recently human papilloma virus, the mechanisms accounting for this association are incompletely defined [1]–[15]. In part, variability in the capacity to detect viruses such as EBV in atherosclerotic lesions has cast doubt on their role in atherogenesis despite epidemiologic and in vitro mechanistic evidence.

The current study supports our previously reported in vitro studies that propose a mechanism linking EBV infection and atherosclerosis. Specifically, we have shown that the early viral protein dUTPase is an important mediator of EBV stimulated inflammation [1], [2], [17], [31], [32], [36].The recognized impact of these pro-inflammatory pathways on atherogenesis as well as the evolution of acute myocardial infarction [18]–[23], [37] suggest a significant role of EBV in the precipitation of acute ischemic events. As a consequence, this prevalent virus likely contributes to the environmental factors that determine coronary artery disease risk.

Deoxyuridine triphosphate nucleotidohyrolase (dUTPase) is an early protein expressed during EBV lytic and abortive-lytic replication [33]. Glaser et al subsequently showed that dUTPase can induce monocytes and macrophages to produce cytokines including IL-6 [31]. In a recent study, Waldmen et al showed that soluble factors produced by dUTPase stimulated monocytes and macrophages induced endothelial cells to increase expression of ICAM-1. They integrated these observations to propose a model by which EBV contributes to the evolution of coronary events [17]. According to this model, dUTPase, which can be encoded and released even during incomplete EBV replication, stimulates monocytes and macrophages by activating NF-κβ through toll like receptor (TLR) 2 to produce pro-inflammatory cytokines, including IL-6 [32]. That these pro-inflammatory cytokines can mediate endothelial damage and the progression of atherosclerosis has been reported in prior investigations [18]–[20], [28]. The dUTPase stimulated monocytes and macrophages further induce endothelial ICAM-1 expression representing activation of the endothelium and promoting the prothrombotic state that leads to acute coronary artery occlusion.

The observations in this clinical study are consistent with the above model. Patients with an acute myocardial infarction had the highest measures of IL-6 and ICAM-1 as has been reported in previous studies [24], [25], [28]–[30], [37]–[39]. However, patients who were dUTPase positive had the highest measures of ICAM-1 and a parallel pattern of IL-6 increase. It is therefore plausible that patients with EBV expressing dUTPase have an augmented stimulation of monocytes and macrophages with increased cytokine production and accelerated endothelial activation reflected in part by greater levels of circulating ICAM-1. dUTPase is an early viral protein and is therefore produced and released even during incomplete viral replication. Because it can be produced even during incomplete viral reactivation, quantities of dUTPase may be released that are sufficient to stimulate a cascade of pro-inflammatory mediators even under conditions of low viral load that are not readily detectable in atherosclerotic lesions. Given the high prevalence of EBV infection, it is plausible that this proposed mechanism contributes to the final, if not earlier, pathways that culminate in an acute myocardial infarction in a significant proportion of patients.

The stimuli known to participate in the reactivation of latent EBV are also known to participate in atherogenesis and the precipitation of acute coronary events. Chief among these are emotional and psychosocial stress that have been shown to dysregulate immune repression of the virus allowing its replication and production of dUTPase [1], [2], [17], [31], [40]. Similarly psychological stress has been shown to be a factor both in the evolution of coronary artery disease and a trigger for acute coronary artery events [41]–[44].

However, the mechanisms and final pathways by which stress events promote atherogenesis and precipitate acute events remain poorly defined. The connection between stress activation of EBV, production of dUTPase, stimulation of pro-inflammatory cytokines, and detectable dUTPase antibody in patients with the most severe coronary artery events and highest levels of IL-6 and ICAM-1 provides an attractive explanation for the connection between stress and coronary artery events. Although likely not the sole link, stress induced EBV reactivation arises as a candidate mediator of stress induced coronary events owing in part to the prevalence of EBV infection.

### Experimental Limitations

The study findings are associative and relate presence of detectable neutralizing antibody against EBV encoded dUTPase to coronary events and corresponding increases in ICAM-1 and IL-6. However, this clinical investigation is consistent with mechanistic models based on in vitro studies and is a necessary next step in confirming the model validity. It provides the impetus for future prospective studies testing the role of EBV as an important risk factor for coronary events.

It is possible that circulating ICAM-1 detected by the methods used in this investigation does not reflect changes in ICAM-1 expression at the cellular level. However, the changes in ICAM-1 measured in this investigation are consistent with the in vitro behavior of endothelial cells exposed to dUTPase stimulated monocytes and macrophages. Further, measures of circulating ICAM-1 have been associated with risk for acute coronary artery events further supporting their measure as a surrogate for cellular ICAM-1 expression [25]–[27].

A variety of factors not yet explored may be important modifiers of the impact of dUTPase on ICAM-1 expression and subsequent coronary events. As an example, a preliminary analysis shows a highly significant interaction between age and dUTPase as determinants of circulating ICAM-1 (data not shown). Specifically, there was an inverse relationship between increasing age and circulating ICAM-1 which was greater in dUTPase positive patients. Interestingly, Il-6 was not found to have this association with patient age. The mechanistic and clinical impact of these preliminary findings as well as the impact of other demographic and environmental factors is unknown and must be explored in future studies.

The data corroborate a pro-inflammatory and pro-atherogenic mechanisms operative in EBV infections. Whether this mechanism plays a role in other viral infections shown to be associated with coronary artery disease must be investigated based on the current findings.

### Conclusions

This investigation provides support for a novel mechanism linking latent viral infections and coronary artery events. The proposed mechanism, which is active even under conditions of low viral load, resolves the conflict between studies demonstrating a causal relation between viral infections and coronary events and those that challenge this relation due to inability to detect virions in atherosclerotic lesions. Further, reactivation of latent EBV may constitute an integral pathway connecting psychological and emotional stress to acute coronary events. These findings provide the support for prospective studies that will continue to investigate the role of latent viral infections as an environmental risk factor for atherosclerosis and acute coronary events, and the capacity of viral infections to serve as mediators of stress induced acute coronary artery syndromes.

## References

[1] Glaser R, Pearson GR, Jones JF, Hillhouse J, Kennedy S, et al (1991) Stress-related activation of epstein-barr virus. Brain Behav Immun 5 (2) 219–232. 1654167 10.1016/0889-1591(91)90018-6

[2] Glaser R, Padgett DA, Litsky ML, Baiocchi RA, Yang EV, et al (2005) Stress-associated changes in the steady-state expression of latent epstein-barr virus: Implications for chronic fatigue syndrome and cancer. Brain Behav Immun 19 (2) 91–103. 15664781 10.1016/j.bbi.2004.09.001

[3] Horvath R, Cerny J, Benedik J Jr, Hokl J, Jelinkova I, et al (2000) The possible role of human cytomegalovirus (HCMV) in the origin of atherosclerosis. J Clin Virol 16 (1) 17–24. 10680737 10.1016/s1386-6532(99)00064-5

[4] Danesh J, Appleby P (1998) Persistent infection and vascular disease: A systematic review. Expert Opin Investig Drugs 7 (5) 691–713. 10.1517/13543784.7.5.691 15991962

[5] Ibrahim AI, Obeid MT, Jouma MJ, Moasis GA, Al-Richane WL, et al (2005) Detection of herpes simplex virus, cytomegalovirus and epstein-barr virus DNA in atherosclerotic plaques and in unaffected bypass grafts. J Clin Virol 32 (1) 29–32. 15572003 10.1016/j.jcv.2004.06.010

[6] Gattone M, Iacoviello L, Colombo M, Castelnuovo AD, Soffiantino F, et al (2001) Chlamydia pneumoniae and cytomegalovirus seropositivity, inflammatory markers, and the risk of myocardial infarction at a young age. Am Heart J 142 (4) 633–640. 11579353 10.1067/mhj.2001.118118

[7] Morre SA, Stooker W, Lagrand WK, van den Brule AJ, Niessen HW (2000) Microorganisms in the aetiology of atherosclerosis. J Clin Pathol 53 (9) 647–654. 11041053 10.1136/jcp.53.9.647 PMC1731245

[8] Stassen FR, Vainas T, Bruggeman CA (2008) Infection and atherosclerosis. An alternative view on an outdated hypothesis. Pharmacol Rep 60 (1) 85–92. 18276989

[9] Stassen FR, Vega-Cordova X, Vliegen I, Bruggeman CA (2006) Immune activation following cytomegalovirus infection: More important than direct viral effects in cardiovascular disease? J Clin Virol 35 (3) 349–353. 16387544 10.1016/j.jcv.2005.11.007

[10] Vliegen I, Herngreen SB, Grauls GE, Bruggeman CA, Stassen FR (2005) Mouse cytomegalovirus antigenic immune stimulation is sufficient to aggravate atherosclerosis in hypercholesterolemic mice. Atherosclerosis 181 (1) 39–44. 15939052 10.1016/j.atherosclerosis.2004.12.035

[11] Lippi G, Franchini M, Favaloro EJ (2010) Influenza and cardiovascular disease: Does swine-origin, 2009 H1N1 flu virus represent a risk factor, an acute trigger, or both? Semin Thromb Hemost 36 (1) 49–58. 20391296 10.1055/s-0030-1248724

[12] Phrommintikul A, Kuanprasert S, Wongcharoen W, Kanjanavanit R, Chaiwarith R, et al (2011) Influenza vaccination reduces cardiovascular events in patients with acute coronary syndrome. Eur Heart J 32 (14) 1730–1735. 21289042 10.1093/eurheartj/ehr004

[13] Warren-Gash C, Smeeth L, Hayward AC (2009) Influenza as a trigger for acute myocardial infarction or death from cardiovascular disease: A systematic review. Lancet Infectious Diseases 9 (10) 601–610. 19778762 10.1016/S1473-3099(09)70233-6

[14] Guan XR, Li X, Xin XM, Jiang LX, Cui LY, et al (2008) Influenza virus infection and risk of acute myocardial infarction. Inflammation 31 (4) 266–272. 18568394 10.1007/s10753-008-9074-2

[15] Dvorakova A, Poledne R (2004) Influenza–a trigger for acute myocardial infarction. Atherosclerosis 172 (2) 391. 15019551 10.1016/j.atherosclerosis.2003.09.005

[16] Pretet JL, Mercier M, Riethmuller D, Aubin F, Vuitton D, et al (2012) Human papillomavirus and cardiovascular disease. J Am Coll Cardiol 60 (1) 81–82. 22742406 10.1016/j.jacc.2012.02.049

[17] Waldman WJ, Williams MV Jr, Lemeshow S, Binkley P, Guttridge D, et al (2008) Epstein-barr virus-encoded dUTPase enhances proinflammatory cytokine production by macrophages in contact with endothelial cells: Evidence for depression-induced atherosclerotic risk. Brain Behav Immun 22 (2) 215–223. 17845840 10.1016/j.bbi.2007.07.007 PMC2245868

[18] Libby P, Ridker PM, Maseri A (2002) Inflammation and atherosclerosis. Circulation 105 (9) 1135–1143. 11877368 10.1161/hc0902.104353

[19] Ross R (1999) Atherosclerosis–an inflammatory disease. N Engl J Med 340 (2) 115–126. 9887164 10.1056/NEJM199901143400207

[20] Fuster V, Badimon L, Badimon JJ, Chesebro JH (1992) The pathogenesis of coronary artery disease and the acute coronary syndromes (2). N Engl J Med 326 (5) 310–318. 1728735 10.1056/NEJM199201303260506

[21] Danesh J, Kaptoge S, Mann AG, Sarwar N, Wood A, et al (2008) Long-term interleukin-6 levels and subsequent risk of coronary heart disease: Two new prospective studies and a systematic review. PLoS Med 5 (4) e78. 18399716 10.1371/journal.pmed.0050078 PMC2288623

[22] Fisman EZ, Benderly M, Esper RJ, Behar S, Boyko V, et al (2006) Interleukin-6 and the risk of future cardiovascular events in patients with angina pectoris and/or healed myocardial infarction. Am J Cardiol 98 (1) 14–18. 16784912 10.1016/j.amjcard.2006.01.045

[23] Lindmark E, Diderholm E, Wallentin L, Siegbahn A (2001) Relationship between interleukin 6 and mortality in patients with unstable coronary artery disease: Effects of an early invasive or noninvasive strategy. JAMA 286 (17) 2107–2113. 11694151 10.1001/jama.286.17.2107

[24] Lee KW, Blann AD, Lip GY (2005) Plasma markers of endothelial damage/dysfunction, inflammation and thrombogenesis in relation to TIMI risk stratification in acute coronary syndromes. Thromb Haemost 94 (5) 1077–1083. 16363252 10.1160/TH05-03-0179

[25] Printseva OY, Peclo MM, Gown AM (1992) Various cell types in human atherosclerotic lesions express ICAM-1. further immunocytochemical and immunochemical studies employing monoclonal antibody 10F3. Am J Pathol 140 (4) 889–896. 1348606 PMC1886365

[26] Empana JP, Canoui-Poitrine F, Luc G, Juhan-Vague I, Morange P, et al (2008) Contribution of novel biomarkers to incident stable angina and acute coronary syndrome: The PRIME study. Eur Heart J 29 (16) 1966–1974. 18621771 10.1093/eurheartj/ehn331

[27] Hwang SJ, Ballantyne CM, Sharrett AR, Smith LC, Davis CE, et al (1997) Circulating adhesion molecules VCAM-1, ICAM-1, and E-selectin in carotid atherosclerosis and incident coronary heart disease cases: The atherosclerosis risk in communities (ARIC) study. Circulation 96 (12) 4219–4225. 9416885 10.1161/01.cir.96.12.4219

[28] Ridker PM, Hennekens CH, Roitman-Johnson B, Stampfer MJ, Allen J (1998) Plasma concentration of soluble intercellular adhesion molecule 1 and risks of future myocardial infarction in apparently healthy men. Lancet 351 (9096) 88–92. 9439492 10.1016/S0140-6736(97)09032-6

[29] Haim M, Tanne D, Boyko V, Reshef T, Goldbourt U, et al (2002) Soluble intercellular adhesion molecule-1 and long-term risk of acute coronary events in patients with chronic coronary heart disease. data from the bezafibrate infarction prevention (BIP) study. J Am Coll Cardiol 39 (7) 1133–1138. 11923036 10.1016/s0735-1097(02)01728-x

[30] Blankenberg S, Rupprecht HJ, Bickel C, Peetz D, Hafner G, et al (2001) Circulating cell adhesion molecules and death in patients with coronary artery disease. Circulation 104 (12) 1336–1342. 11560847 10.1161/hc3701.095949

[31] Glaser R, Litsky ML, Padgett DA, Baiocchi RA, Yang EV, et al (2006) EBV-encoded dUTPase induces immune dysregulation: Implications for the pathophysiology of EBV-associated disease. Virology 346 (1) 205–218. 16321417 10.1016/j.virol.2005.10.034

[32] Ariza ME, Glaser R, Kaumaya PT, Jones C, Williams MV (2009) The EBV-encoded dUTPase activates NF-kappa B through the TLR2 and MyD88-dependent signaling pathway. J Immunol 182 (2) 851–859. 19124728 10.4049/jimmunol.182.2.851 PMC12892303

[33] Williams MV, Parris DS (1987) Characterization of a herpes simplex virus type 2 deoxyuridine triphosphate nucleotidohydrolase and mapping of a gene conferring type specificity for the enzyme. Virology 156 (2) 282–292. 3027979 10.1016/0042-6822(87)90408-9

[34] Williams MV, Holliday J, Glaser R (1985) Induction of a deoxyuridine triphosphate nucleotidohydrolase activity in epstein-barr virus-infected cells. Virology 142 (2) 326–333. 2997989 10.1016/0042-6822(85)90341-1

[35] Jones JF, Williams M, Schooley RT, Robinson C, Glaser R (1988) Antibodies to epstein-barr virus-specific DNase and DNA polymerase in the chronic fatigue syndrome. Arch Intern Med 148 (9) 1957–1960. 2843138

[36] Padgett DA, Hotchkiss AK, Pyter LM, Nelson RJ, Yang E, et al (2004) Epstein-barr virus-encoded dUTPase modulates immune function and induces sickness behavior in mice. J Med Virol 74 (3) 442–448. 15368518 10.1002/jmv.20196

[37] Lee KW, Lip GY, Tayebjee M, Foster W, Blann AD (2005) Circulating endothelial cells, von willebrand factor, interleukin-6, and prognosis in patients with acute coronary syndromes. Blood 105 (2) 526–532. 15374879 10.1182/blood-2004-03-1106

[38] Postadzhiyan AS, Tzontcheva AV, Kehayov I, Finkov B (2008) Circulating soluble adhesion molecules ICAM-1 and VCAM-1 and their association with clinical outcome, troponin T and C-reactive protein in patients with acute coronary syndromes. Clin Biochem 41 (3) 126–133. 18061588 10.1016/j.clinbiochem.2007.09.001

[39] Rohde LE, Hennekens CH, Ridker PM (1999) Cross-sectional study of soluble intercellular adhesion molecule-1 and cardiovascular risk factors in apparently healthy men. Arterioscler Thromb Vasc Biol 19 (7) 1595–1599. 10397675 10.1161/01.atv.19.7.1595

[40] Kiecolt-Glaser JK, Dura JR, Speicher CE, Trask OJ, Glaser R (1991) Spousal caregivers of dementia victims: Longitudinal changes in immunity and health. Psychosom Med 53 (4) 345–362. 1656478 10.1097/00006842-199107000-00001

[41] Dimsdale JE (2008) Psychological stress and cardiovascular disease. J Am Coll Cardiol 51 (13) 1237–1246. 18371552 10.1016/j.jacc.2007.12.024 PMC2633295

[42] Bhattacharyya MR, Steptoe A (2007) Emotional triggers of acute coronary syndromes: Strength of evidence, biological processes, and clinical implications. Prog Cardiovasc Dis 49 (5) 353–365. 17329181 10.1016/j.pcad.2006.11.002

[43] Rozanski A, Blumenthal JA, Davidson KW, Saab PG, Kubzansky L (2005) The epidemiology, pathophysiology, and management of psychosocial risk factors in cardiac practice: The emerging field of behavioral cardiology. J Am Coll Cardiol 45 (5) 637–651. 15734605 10.1016/j.jacc.2004.12.005

[44] Emani S, Binkley PF (2010) Mind-body medicine in chronic heart failure: A translational science challenge. Circ Heart Fail 3 (6) 715–725. 21081739 10.1161/CIRCHEARTFAILURE.110.951509

